# Isolated unilateral brachial plexus injury following carbon monoxide intoxication: a case report and literature review

**DOI:** 10.3389/fneur.2024.1346353

**Published:** 2024-05-09

**Authors:** Shu Liu, Houchao Sun, Shengyuan Wang, Jincheng Liao, Xu Yang, Zhiyou Cai

**Affiliations:** ^1^Chongqing Medical University, Chongqing, China; ^2^Chongqing Institute of Green and Intelligent Technology, Chinese Academy of Sciences, Chongqing, China; ^3^Chongqing School, University of Chinese Academy of Sciences, Chongqing, China; ^4^Department of Neurology, Chongqing General Hospital, Chongqing, China; ^5^Chongqing Key Laboratory of Neurodegenerative Diseases, Chongqing University, Chongqing, China

**Keywords:** carbon monoxide intoxication, peripheral neuropathy, hyperbaric oxygen therapy, spinal cord ischemia, rehabilitation training

## Abstract

Carbon monoxide (CO) is a gas that has no odor or color, making it difficult to detect until exposure leads to coma or death. CO poisoning is one of the most common and deadly poisonings around the world. CO poisoning is a common and often fatal form of poisoning worldwide. A toxic effect of CO is tissue hypoxia, which leads to systemic complications. Additionally, there may be severe neurological symptoms and delayed complications following CO poisoning. However, peripheral neuropathy is relatively rare after CO poisoning. Previously, only one case of unilateral plexopathy after CO poisoning, accompanied by rhabdomyolysis and cognitive dysfunction, has been reported. In this report, an isolated unilateral brachial plexopathy following CO intoxication is described. A key mechanism in this case may be CO-induced spinal cord ischemia. Immediate administration of hyperbaric oxygen therapy (HBOT) is crucial to prevent peripheral neuropathy after acute CO intoxication. Hyperbaric oxygen therapy (HBOT) should be administered immediately after acute CO intoxication to prevent peripheral neuropathy. Additionally, peripheral neuropathy following acute CO intoxication may benefit from consistent rehabilitation training.

## Introduction

Carbon monoxide (CO) is a gas that is both colorless and odorless, making it difficult to detect. Its potential danger lies in its ability to cause coma or even death without being noticed. CO poisoning is a common and fatal type of poisoning that is widespread across the globe. The toxicity of CO leads to tissue hypoxia, resulting in a range of systemic and neurological complications. However, the occurrence of peripheral neuropathy following CO poisoning is relatively uncommon and typically affects younger individuals.

## Case report

On 11 February 2022, a 31-year-old man was found unconscious by his wife after sleeping in a closed car for approximately 7 h. He was admitted to the hospital and diagnosed with acute CO poisoning. Blood tests showed an elevated level of carbon monoxide hemoglobin (13.8%). Brain magnetic resonance imaging (MRI) revealed small, slightly brighter signals in the bilateral basal ganglia. After being in a coma for about 12 h, he regained consciousness without any cognitive deficits. However, he woke up with weakness in his left upper arm and limited movement of the limb.

**Figure 1:**
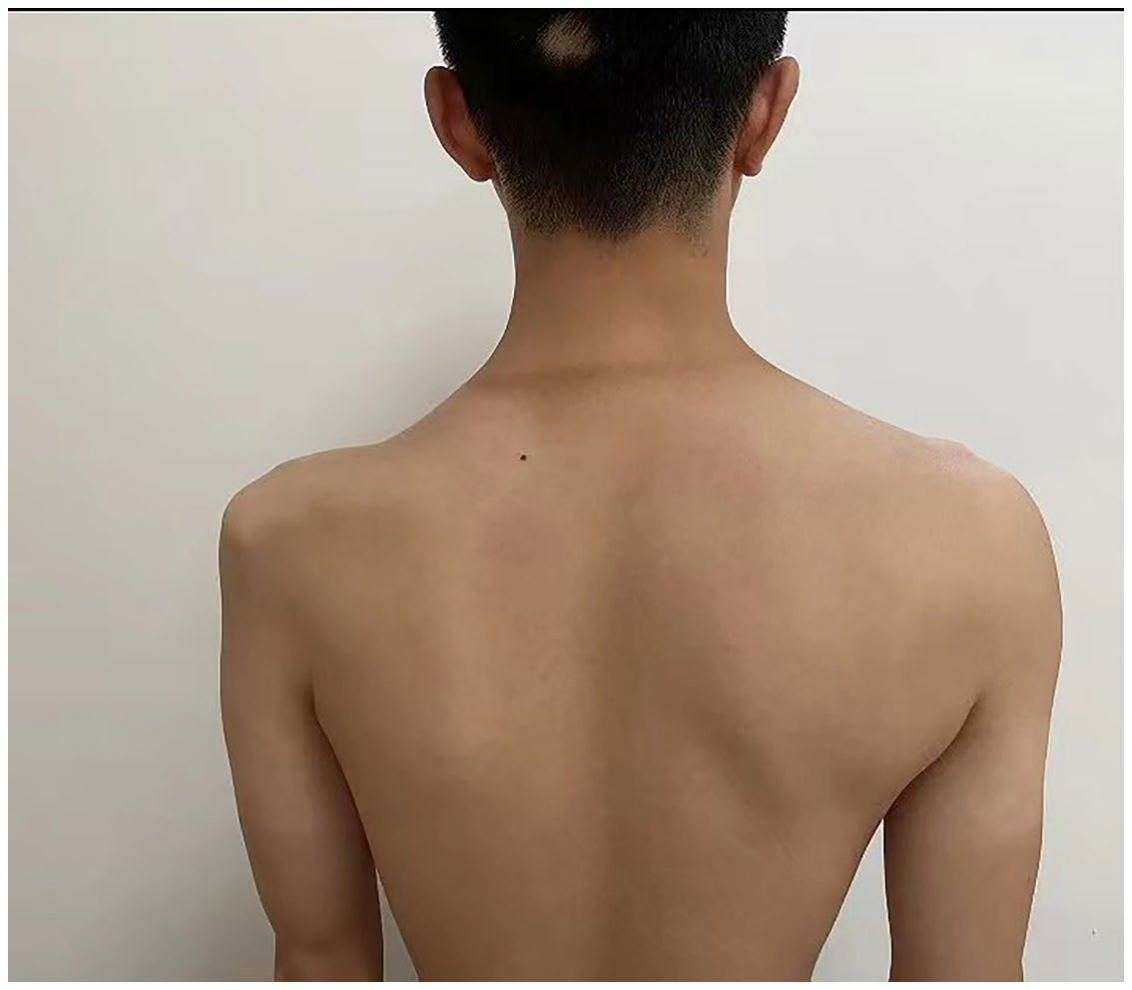
The muscle profile of the patient.

The patient’s left arm was paralyzed, as indicated by a grade 1 muscle test on the medical research council (MRC) scale during neurological examination. There was also reduced sensation to temperature, pain, and vibration in the distal region of the left arm, and absent deep tendon reflexes in the left upper arm. Ultrasonic examination showed swelling of the left median and radial nerves in the forearm and upper arm. A motor nerve conduction study indicated normal motor conduction velocity (MCV) for the left ulnar, median, and radial nerves, but a decreased compound muscle action potentials (CMAP) amplitude for the left axillary and musculocutaneous nerves. Sensory conduction velocity (SCV) for the left median and ulnar nerves fell within the normal range (Table 1). Needle electromyography revealed abnormal spontaneous activities, including positive sharp waves and fibrillation potentials, in the left deltoid, biceps brachii, and triceps brachii (Table 2). Electrophysiological interpretation suggested severe abnormalities in the upper branches and moderate abnormalities in the middle branches, indicating the presence of left brachial plexopathy involving the upper and middle branches. After receiving around 80 sessions of hyperbaric oxygen therapy (HBOT) once daily for 90 min each session, and undergoing rehabilitation training including exercise training, low-frequency pulsed electrical therapy, ultrasound therapy, as well as acupuncture, massage, electroacupuncture, and acupoint injection, the patient returned to the hospital for a follow-up examination 70 days after the CO poisoning incident. There was a slight improvement in the restricted movement of his left upper limb. Manual muscle tests revealed an MRC grade of 3 in the muscle groups of the left upper arm, including the left biceps brachii, triceps brachii, brachialis, brachioradialis, and deltoid. Sensation to pinprick and light touch in the left upper limb was present. Additionally, the patient did not show any other long-term effects. However, significant atrophy of the left deltoid and biceps muscles was noted (Figure 1). Compared to the initial admission, the re-examination of the cerebral MRI showed no significant changes. The sensory nerve conduction study revealed decreased nerve conduction velocity and amplitude in the left radial nerves (Table 3). Motor nerve conduction was normal, except for highly decreased motor amplitude in the left axillary and musculocutaneous nerves (Table 4). Besides, the N20 latency of the left median nerve is normal, but the amplitude is relatively lower compared to the right side, suggesting abnormal left upper limb somatosensory evoked potentials (SEP). The electrophysiological conclusion for this readmission indicated left incomplete brachial plexopathy.

**Table 1:** Motor nerve conduction study and sensory nerve conduction study in the patient after carbon monoxide intoxication.

Nerve	Dl	Amp	Dis	Vel
	mS	mV	mm	m/s
**MNCS**				
**Axillaris left**				
Erb-Del.	8.85	1.19		
**Medianus left**				
Erb-Del.	3.67	12.5		
**Medianus left**				
Wrist-APB	3.27	20.1		
Elb-APB	6.72	18.1	195	56.5
Elb-F	25.8	0.55		
**Musculocutaneous left**				
Erb-Biceps	8.41	0.29		
**Musculocutaneous right**				
Erb-Tricep	4.67	24.8		
**Radialis left**				
Erb-Tricep	4.45	19.7		
**Ulnaris left**				
Wrist-ADM	2.42	13.1		
Elbow-ADM	5.33	12.9	195	67
Stim 1-F	21.1	2.3		
Stim 2-F	20.3	2.7		
**SNCS**				
**Medianus left**				
Wrist-DigII	2.6	17.6	170	65.4
**Ulnaris left**				
Wrist-DigV	1.96	46.9	120	61.2

MNCS, motor nerve conduction study; Dl, distal latency; Amp, amplitude; Dis, distance; Vel, velocity; Erb, elbow; Del, deltoid; APB, abductor pollicis brevis; ADM, abductor digiti minimi; Sti, stimulation; SNCS, sensory nerve conduction study.

**Table 2:** Electromyographic findings in the patient after carbon monoxide intoxication.

Spontaneous activities			
Muscle	Explanation	Fib	PSW
Right Biceps	Normal	0/10	0/10
Right Deltoid	Normal	0/10	0/10
Right Vastus	Normal	0/10	0/10
Left Vastus	Normal	0/10	0/10
Left ADM	Normal	0/10	0/10
Left Abd pollicis brevis	Normal	0/10	0/10
Left Triceps		2/10	0/10
Left Biceps	Loss of MU	6/10	6/10
Left Deltoid	Loss of MU	6/10	6/10

Fib, fibrillation potential; PSW, positive sharp wave; ADM, abductor digiti minimi; Abd, abductor.

**Table 3:** Sensory nerve conduction study in the patient when readmission.

Nerve/Part	Site	Onset Lat	Peak Lat	Amp	Distance	Velocity
		ms	ms	mm	mm	m/s
**Left median nerve—middle finger**						
Wtist	DigIII	2.4	3.18	56.4	125	52
**Left ulnar nerve—Little finger**						
Wrist	DigV	2.08	2.81	31.5	100	48
**Left radial nerve—anatomical snuff box (Forearm)**						
Forearm	Wrist	2.19	2.92	14.0	95	43

Lat, latency; Dig, digiti; Amp, amplitude.

**Figure 2:**
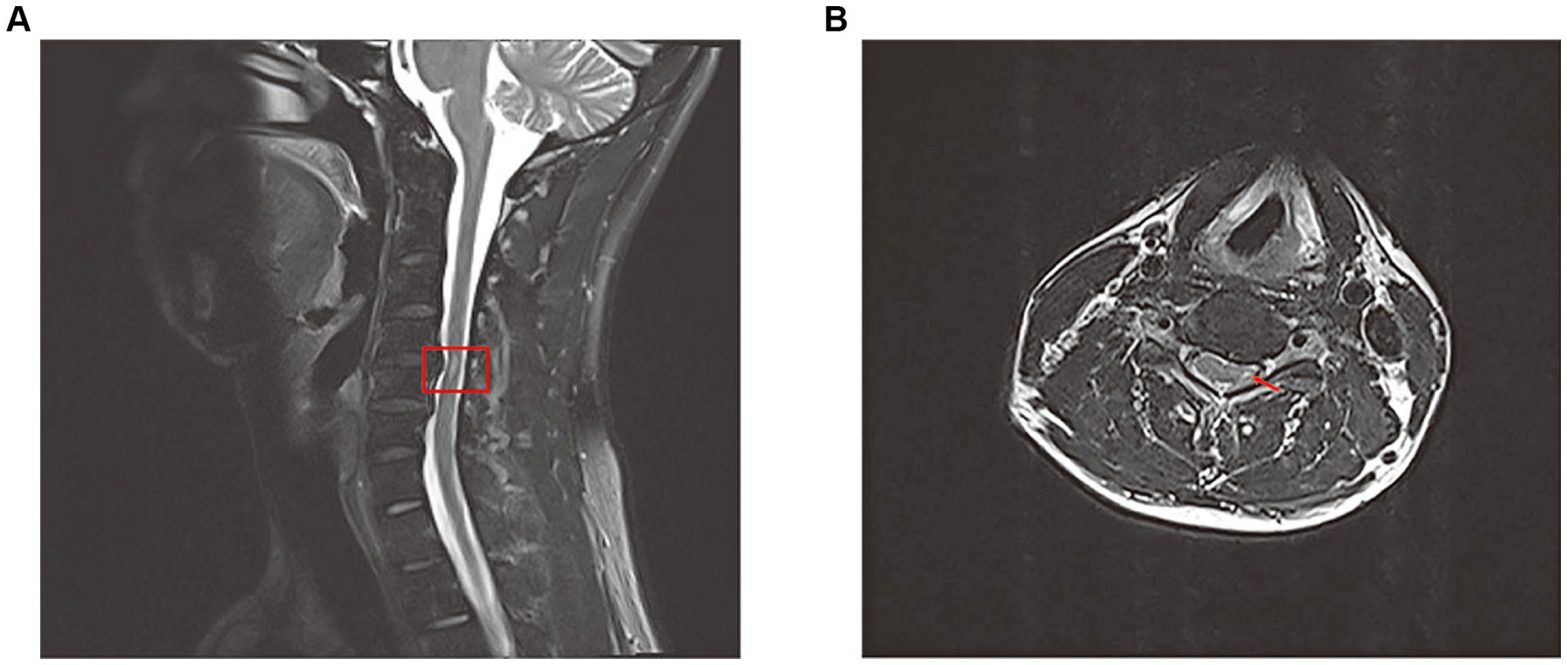
Total spine magnetic resonance image(MRI) revealed long strip T2 high signal in the left part of the cervical medulla at the level of the 4/5 intervertebral space on sagittal position **(A)** and cross section **(B)**.

The patient’s subsequent examination findings remained largely unchanged from the initial assessment. However, we conducted a comprehensive magnetic resonance imaging (MRI) of the entire spine for the patient. The results revealed a long strip of high signal intensity in the left region of the cervical medulla at the 4/5 intervertebral space level (Figure 2). Throughout his current hospital stay, the patient has been receiving ongoing rehabilitation training and HBOT.

**Table 4:** Motor nerve conduction study in the patient when readmission.

Nerve/Part	Muscle	Latency	Amplitude	Distance	Velocity
		ms	mV	mm	m/s
**Left radial nerve—EIP**					
Forearm	EIP	2.34	3.3		
Spiral Gr	EIP	3.7	3.6	80	59
**Left median nerve—Abductor pollicis brevis**					
Wrist	Abductor pollicis brevis	3.23	9.4		
Elbow	Abductor pollicis brevis	6.25	10.9	160	53
**Left ulnar nerve—ADM**					
Wrist	Abductor digiti minimi	2.66	10.9		
Elbow	Abductor digiti minimi	5.1	9.0	160	65
**Left axillary nerve—Deltoid**					
Erb's	Deltoid	2.92	8.9	190	
**Left musculocutaneous nerve—Biceps**					
Erb's	Biceps	4.79	1.6	302	
**Right axillary nerve—Deltoid**					
Erb's	Deltoid	3.44	28		
**Right musculocutaneous nerve—Biceps**					
Erb's	Biceps	3.96	9.1		
**Right radial nerve—EIP**					
Forearm	EIP	2.60	8.9		
Spiral Gr	EIP	4.01	9.2	95	68

EIP, extensor indicis proprius; Erb, elbow; ADM, abductor digiti minimi.

## Discussion

Carbon monoxide (CO) is known to have toxic effects on various systems in the body, including the central nervous system, cardiovascular system, respiratory system, hematological system, metabolic and endocrinological system, musculoskeletal system, and sensory systems such as vision and hearing. While there have been numerous reports on the effects of CO poisoning on the cardiovascular system and central nervous system, such as memory loss, encephalopathy, speech difficulties and Parkinson’s-like symptoms, however, there is a limited amount of research available specifically focusing on the development of peripheral neuropathies in CO poisoning cases.

To the best of our knowledge, a comprehensive clinical study on peripheral neuropathy following CO poisoning has been conducted. The study examined 2,759 patients with acute CO poisoning between 1976 and 1982, and 23 individuals (11 men and 12 women, with a mean age of 29.3) were diagnosed with peripheral neuropathy through electrophysiological detection. Among these patients, 14 had sensory symptoms, 8 had mixed symptoms, and only one had isolated motor symptoms. The majority of cases involved the lower extremities, with only two cases affecting other areas. The study also revealed that peripheral neuropathy following CO poisoning primarily affected young individuals, and all patients recovered within a period of 3 to 6 months (1). Furthermore, Rahmani et al. (2) reported a case of a 42-year-old man who experienced reversible bilateral brachial plexus injury following acute CO poisoning. The patient showed a favorable prognosis after receiving HBOT. Gi-Young Park et al. (3) reported a case of unilateral brachial plexopathy accompanied by impaired cognitive function after CO intoxication. Electrophysiological interpretation indicated an incomplete brachial plexopathy with axonal involvement. The 45-year-old patient exhibited a poor prognosis, with no significant improvement in left upper extremity weakness and cognitive function even after 11 months of CO intoxication. Other articles have also documented cases of peripheral neuropathy following CO intoxication, including sciatic neuropathy and rhabdomyolysis, motor and sensory peripheral neuropathy, and unilateral diaphragmatic paralysis.

**Figure 3:**
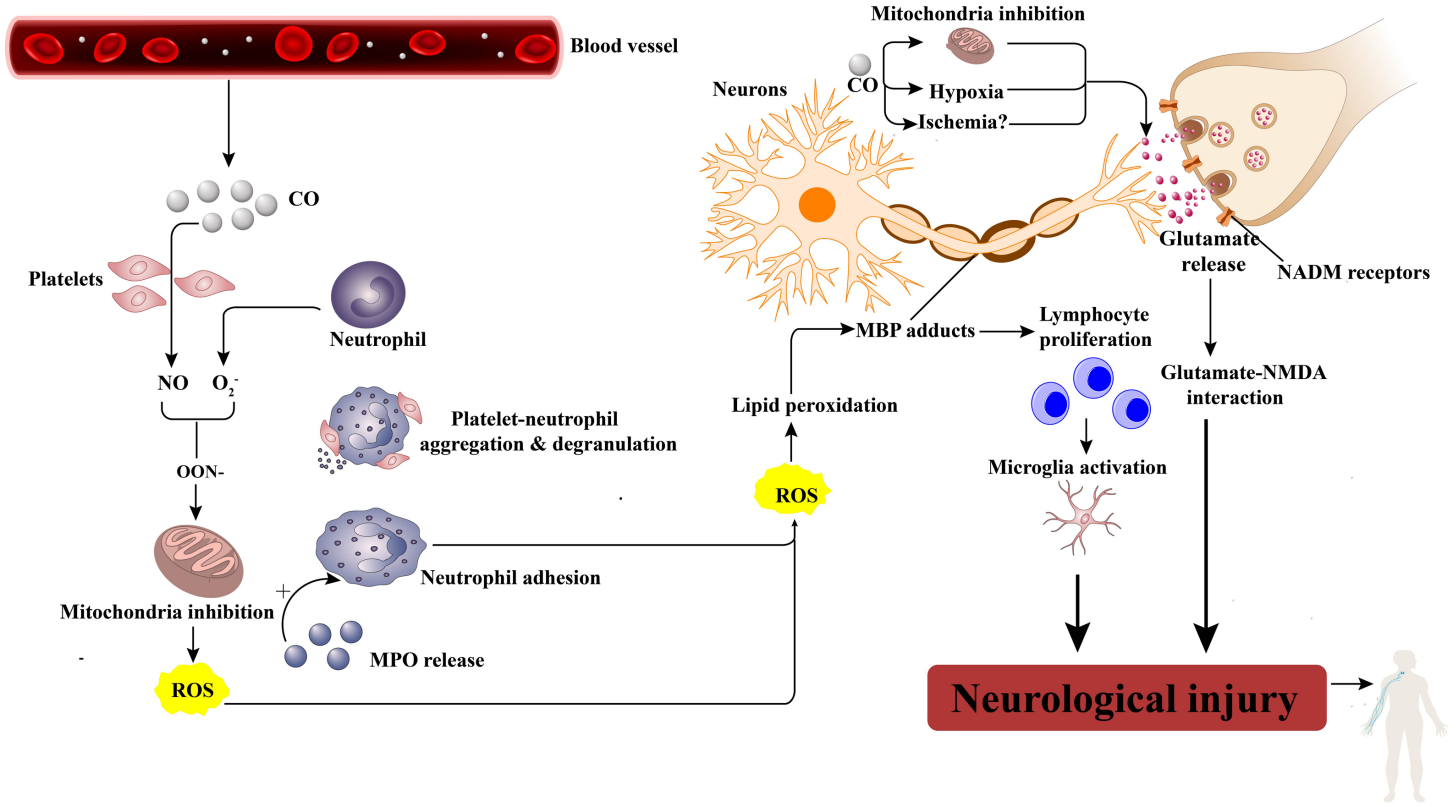
CO leads to neurologic injury. CO activates platelets by displacing platelet nitric oxide (NO) from surface hemoproteins. NO reacts with oxygen free radicals (O2^−^) to produce peroxynitrite (ONOO2), which inhibits mitochondrial function and produces reactive oxygen species(ROS). Besides, activated neutrophils by NO will degranulate and release myeloperoxidase (MPO), which causes more neutrophil activation and adhesion, thereby causing cellular damage as well as lipid peroxidation, specifically on myelin basic protein (MBP). When peroxidated, MBP forms adducts that cause lymphocyte proliferation, microglia activation, and, finally, neurologic injury. Moreover, the general effects of hypoxia and the effect of CO toxicity directly on mitochondria cause glutamate release, which activates N-methyl-D-aspartate (NMDA) receptors, further leading to neurologic injury.

The exact underlying mechanisms of peripheral neuropathy following CO poisoning are still uncertain. However, there are four potential mechanisms that could contribute to this condition: hypoxia and subsequent ischemia caused by CO, nerve compression, the cytotoxic effects of CO, and petechial hemorrhages. CO induces hypoxia by forming carboxyhemoglobin (COHb) and binding to heme-containing proteins, particularly cytochrome c oxidase and myoglobin. Additionally, CO binds to heme proteins in platelets, leading to the release of nitric oxide (NO). Increased levels of NO result in the production of peroxynitrite (ONOO), which impairs mitochondrial function and exacerbates tissue hypoxia (Figure 3) (4). The central nervous system is particularly susceptible to the effects of ischemia and hypoxia. The thoracic spinal cord receives blood supply from the radiculomedullary arteries, which originate from a few intercostal arteries branching from the subclavian artery and aorta. Impairment of blood flow through these arteries can pose a significant risk of ischemia due to limited collateralization in the thoracic vascular region. Additionally, the watershed effect, which occurs when two streams of blood flowing in opposite directions meet, is common in the spinal cord’s vascular system. The midthoracic area exhibits the maximum watershed effect due to the greatest distance between radicular arteries (5). In this case, a long strip of high T2 signal was observed in the left part of the cervical medulla at the level of the 4/5 intervertebral space. An abnormal signal intensity was also present in the left cervical spinal cord, correlating with the clinical symptoms and signs. A disk protrusion was noted at that level, although it did not compress the spinal cord sufficiently to cause brachial plexus injury. The patient was found lying in the driver’s seat with his head tilted to the left by his wife. It is possible that spondylogenic compression myelopathy (possibly due to ischemia) may have been related to the unconscious patient’s position in the car. Therefore, we suspect that spinal cord ischemia caused by CO intoxication may be a key mechanism in the development of left brachial plexopathy. Additionally, nerve compression and the cytotoxic effects of CO should also be taken into consideration.

In our case, a young man experienced isolated unilateral brachial plexus injury following acute CO poisoning, with incomplete improvement after nearly 3 months. This represents the second reported case of unilateral brachial plexopathy following CO intoxication in the literature thus far.

## Conclusion

The main mechanism behind unilateral brachial plexopathy following CO poisoning may be the ischemia of the spinal cord. Therefore, it is crucial to promptly address the hypoxia caused by CO through treatments like HBOT and normobaric 100% oxygen therapy to prevent peripheral neuropathy after acute CO intoxication. Additionally, consistent participation in rehabilitation training may play a significant role in the treatment of peripheral neuropathy following acute CO poisoning.

## Data availability statement

The original contributions presented in the study are included in the article/supplementary material, further inquiries can be directed to the corresponding author.

## Ethics statement

The studies involving humans were approved by the Institutional Review Board at Chongqing General Hospital. The studies were conducted in accordance with the local legislation and institutional requirements. Written informed consent for participation was not required from the participants or the participants’ legal guardians/next of kin in accordance with the national legislation and institutional requirements. Written informed consent was obtained from the individual(s) for the publication of any potentially identifiable images or data included in this article.

## Author contributions

SL: Conceptualization, Formal analysis, Visualization, Writing – original draft, Writing – review & editing. HS: Formal analysis, Writing – review & editing. SW: Formal analysis, Visualization, Writing – review & editing. JL: Visualization, Writing – review & editing. XY: Visualization, Writing – review & editing. ZC: Funding acquisition, Supervision, Writing – review & editing.

## References

[1] Choi IS. A clinical study of peripheral neuropathy in carbon monoxide intoxication. Yonsei Med J. (1982) 23:174–7. doi: 10.3349/ymj.1982.23.2.174, PMID: 6314683

[2] Rahmani M Belaidi H Benabdeljlil M Bouchhab W El Jazouli N El Brini A. Bilateral brachial plexus injury following acute carbon monoxide poisoning. BMC Pharmacol Toxicol. (2013) 14:61. doi: 10.1186/2050-6511-14-61, PMID: 24314014 PMC3866568

[3] Park GY Kwon DR Jung WB. Unilateral brachial plexus injury following carbon monoxide intoxication: a case report. Medicine. (2018) 97:e11699. doi: 10.1097/MD.0000000000011699, PMID: 30045328 PMC6078647

[4] Chenoweth JA Albertson TE Greer MR. Carbon Monoxide Poisoning. Crit Care Clin. (2021) 37:657–72. doi: 10.1016/j.ccc.2021.03.010 34053712

[5] Martirosyan NL Feuerstein JS Theodore N Cavalcanti DD Spetzler RF Preul MC. Blood supply and vascular reactivity of the spinal cord under normal and pathological conditions. J Neurosurg Spine. (2011) 15:238–51. doi: 10.3171/2011.4.SPINE10543 21663407

